# Sodium Lactate Accelerates M2 Macrophage Polarization and Improves Cardiac Function after Myocardial Infarction in Mice

**DOI:** 10.1155/2021/5530541

**Published:** 2021-06-05

**Authors:** Jialiang Zhang, Fangyang Huang, Li Chen, Guoyong Li, Wenhua Lei, Jiahao Zhao, Yanbiao Liao, Yijian Li, Changming Li, Mao Chen

**Affiliations:** Department of Cardiology, West China Hospital, Sichuan University, Chengdu, China

## Abstract

**Background:**

After myocardial infarction, anti-inflammatory macrophages perform key homeostatic functions that facilitate cardiac recovery and remodeling. Several studies have shown that lactate may serve as a modifier that influences phenotype of macrophage. However, the therapeutic role of sodium lactate in myocardial infarction (MI) is unclear.

**Methods:**

MI was established by permanent ligation of the left anterior descending coronary artery followed by injection of saline or sodium lactate. Cardiac function was assessed by echocardiography. The cardiac fibrosis area was assessed by Masson trichrome staining. Macrophage phenotype was detected via qPCR, flow cytometry, and immunofluorescence. Signaling proteins were measured by Western blotting.

**Results:**

Sodium lactate treatment following MI improved cardiac performance, enhanced anti-inflammatory macrophage proportion, reduced cardiac myocytes apoptosis, and increased neovascularization. Flow-cytometric analysis results reported that sodium lactate repressed the number of the IL-6+, IL-12+, and TNF-*α*+ macrophages among LPS-stimulated bone marrow-derived macrophages (BMDMs) and increased the mRNA levels of Arg-1, YM1, TGF-*β*, and IL-10. Mechanistic studies revealed that sodium lactate enhanced the expression of P-STAT3. Furthermore, a STAT3 inhibitor eliminated sodium lactate-mediated promotion macrophage polarization.

**Conclusion:**

Sodium lactate facilitates anti-inflammatory M2 macrophage polarization and protects against MI by regulating P-STAT3.

## 1. Introduction

Ischemic heart disease is the most common cause of death, and the frequency is increasing worldwide. Even though acute treatments and long-term care improve the clinical presentation of acute coronary syndrome (ACS), heart disease is still the most common cause of death in the word, and the treatment is also a challenge [1–3]. Accumulating evidence indicates that inflammatory reactions are essential events in the healing process and cardiac postinfarction remodeling. Cardiac repair after myocardial infarction (MI) is initiated by robust tissue inflammation, followed by active suppression and resolution. Numerous cellular and molecular factors influence post-MI wound healing and repair [4]. Tissue regeneration is highly dependent on the phenotype or polarization state of a macrophage. In addition, macrophages are generally classified into two main phenotypes, proinflammatory (M1) and anti-inflammatory macrophages (M2). M1 macrophages secrete various proinflammatory cytokines such as IL-1*β*, IL-12, IL-1*α*, IL-6, IL-23, TNF-*α*, and cyclooxygenase-2 (COX-2), all of which contribute to inflammation. In contrast, M2 macrophages are downregulating inflammatory cytokines and upregulating anti-inflammatory cytokines such as IL-12 and high amounts of IL-10, TGF-*β*, and VEGF that promote anti-inflammatory effects and repair [5, 6]. In the early period post-MI, the injured heart is dominated by proinflammatory macrophages, several days later, the anti-inflammatory macrophages take over. Moreover, the death of cardiomyocytes releases damage-associated molecular patterns (DAMPs), such as fibronectin fragments, nuclear chromatin-binding protein high mobility group box 1, low molecular hyaluronic acid, cardiac myosin, mitochondrial DNA and circulating extracellular RNA molecules, and heat shock proteins, resulting in a systemic inflammatory response and a local reaction to recruit circulating monocytes into the infarcted site [7–9]. Therefore, macrophages play a very important role in the initiation, development, and resolution of the immune response following MI. Macrophages also display functional heterogeneity that guides proper wound healing. Efficient clearance of apoptotic cells and a reparative fibrotic response are essential for tissue repair after MI. However, these progresses are orchestrated by M2 macrophages [9–11]. Furthermore, targeting macrophage polarization is a novel therapeutic strategy for MI treatment.

Lactate was previously considered as a metabolic by-product, and accumulating evidence has shown that lactate is critical for metabolic regulation and plays a pivotal role in regulating diverse biological processes [12, 13]. Colegio et al. found that tumor-associated macrophage (TAM) polarization is dependent on tumor-derived lactic acid, and the mechanism is mediated by hypoxia-inducible factor 1*α* (HIF1*α*) [14]. Furthermore, several studies also showed that lactic acid was sufficient to induce macrophage polarization in a manner that depended on an acidic pH in several cancer microenvironments [15–17], and the underline mechanism was undetermined. Additionally, the cellular response to lactate in the tumor microenvironment is quite different from that occurring in the context of chronic inflammation. Therefore, new studies on this context present multiple potential clinical applications. Moreover, more recent evidence has revealed that macrophage development, phenotype, and function demonstrate remarkable heterogeneity and plasticity in heart and other tissues [9, 10, 18]. Thus, whether sodium lactate participates in macrophage polarization under inflammatory conditions and whether sodium lactate has a potential therapeutic effect on MI remain unclear. In our study, we found that sodium lactate accelerated the polarization of bone marrow-derived macrophages (BMDMs) in the presence of lipopolysaccharide (LPS). Furthermore, sodium lactate also provided cardioprotective effects via promoting macrophage polarization in a MI mouse model.

## 2. Materials and Methods

### 2.1. Animals and Treatments

All animal experiments were conducted according to the guidelines on the use and care of laboratory animals for biomedical research published by the National Institutes of Health (No. 85-23, revised 1996) and approved by the committee on the Ethics of Animal Experiments of West China Hospital (No. 2019189A), Sichuan University. Male C57BL/6 mice aged 8 to 10 weeks were anaesthetized with 3% isoflurane. Then, mice were placed in the right lateral decubitus position. With the aid of surgical microscope, the left anterior descending artery (LAD) could be visualized and permanently ligated with a 7-0 silk suture at the site of 2 mm below the left appendix. Sodium lactate (2 g/Kg/day. Sigma-Aldrich, USA) or saline was treated intraperitoneally one day after operation.

### 2.2. Histological Preparation

On day 3 or day 14 after treatment with sodium lactate or saline, myocardial infarction (MI) mice were anaesthetized and the hearts were exposed. For pathological examinations, the hearts were arrested via ventricular injection of 1 ml of 1 mol/L KCl followed by 5 ml PBS and 10 ml 4% paraformaldehyde perfusion. Then, the hearts were carefully dissected and fixed in 4% paraformaldehyde overnight. For biochemical and molecular analysis, the hearts were perfused with PBS and then snap-frozen in liquid nitrogen and stored at -80°C.

### 2.3. Flow Cytometry

To assess macrophage polarization 3 days after MI, 8–10-week-old male mice were anaesthetized, intracardially perfused with ice-cold PBS to exclude the blood cells. Then, the heart was dissected, enzymatically digested with a cocktail of 125 U/ml collagenase XI, 60 U/ml DNase I, 450 U/ml collagenase I, and 60 U/ml hyaluronidase (Sigma-Aldrich) for 1 h at 37°C with gentle agitation. After digestion, the tissue was triturated and passed through a 70 *μ*m cell strainer. Leukocyte-enriched fractions were isolated by 37–70% Percoll (GE Healthcare) density gradient centrifugation. Data were analyzed with a FACSCalibur instrument (BD Biosciences, San Jose, CA, USA) using the NovoExpress software, gating strategies to detect different subsets of infiltrating immune cells. Immune cells were gated on a forward scatter (FSC)/side scatter (SSC) plot. The live cells were further gated to determine leukocytes (CD45+ cells) and then further gated to determine monocytes (CD11b+Ly6Glow) and macrophages (CD11b+Ly6G-F4/80+). The CD11b+ cells were then further gated to determine M1 (F4/80+CD206low) and M2 (F4/80+CD206high) macrophages. The antibodies used were obtained from BioLegend.

### 2.4. Echocardiography

Echocardiography analysis was performed 14 days after IR surgery. Mice were anaesthetized with isoflurane, and cardiac function was assessed using an echocardiography machine (Vevo3100, FUJIFILM). Images were acquired of the left ventricle to measure the situation of cardiac function.

### 2.5. Histochemistry and Immunofluorescence

Histological staining for immunohistochemistry was performed on heart tissue that had been harvested, fixed in 4% paraformaldehyde, embedded in paraffin, sliced into sections, and blocked with 5% BSA for 0.5 h, after which the sections were washed with PBS. The samples were then covered with 5% BSA for 0.5 h and incubated with antibodies (anti-CD31, 1 : 200; Abcam, anti-Arg-1, 1 : 100; ProteinTech Group, anti-CD206 1 : 100; ProteinTech Group) at 4°C overnight. For histochemistry, the samples were then immunostained with HRP-conjugated antibodies (Abcam) for 4 h at room temperature. For immunofluorescence, after the tissue incubated with antibody overnight and then washed three times with PBS, the sections were incubated with appropriate secondary antibody Alexa Fluor 594 AffiniPure donkey anti-rat for 2 hours at room temperature. After 3 times additional washing with PBS, the sections were sealed with DAPI Fluoromount-GTM (Yeasen) to identify nuclei. Fluorescence microscopy images were caught with microscope (IX83; Zeiss). Masson's trichrome staining was performed for cardiac fibrosis analysis. Staining was observed using fluorescence microscope (IX83; Zeiss). Quantification of all data was performed with the ImageJ software.2.2.

### 2.6. Terminal Deoxynucleotidyl Transferase dUTP Nick End Labelling (TUNEL) Staining

Heart tissue was harvested, fixed in 4% paraformaldehyde, embedded in paraffin, sliced into sections, and blocked with 5% BSA for 0.5 h, after which the sections were washed with PBS, sectioned into 3 *μ*m slices, permeabilized with 0.3% Triton X-100 for 20 minutes, and blocked for 30 minutes with 5% bovine serum albumin. It was stained using a TUNEL FITC Apoptosis Detection Kit (Beyotime Biotechnology, China) according to the manufacturer's instructions. Nuclei were counterstained with 4′,6-diamidino-2-phenylindole (DAPI, 1 *μ*g/ml). Finally, images were captured with a Zeiss IX83 microscope.

### 2.7. Cell Culture and Drug Treatment

The bone marrow-derived macrophages (BMDMs) used in this study were obtained from male 8-10-week-old mouse bone marrow. In brief, the mice were sacrificed; their femurs and tibias were removed after alcohol disinfection; the bone marrow was collected, filtered through a 70 *μ*m strainer (BD falcon), and centrifuged for 5 min at 1000 rpm; the cells obtained were cultured on petri dishes in RPMI 1640 medium (HyClone; GE Healthcare Life Sciences) supplemented with 10% fetal bovine serum (FBS; Gibco; Thermo Fisher Scientific, Inc), 20 ng/ml macrophage colony stimulating factor (M-CSF; PeproTech; 400-28), and 1% penicillin/streptomycin (HyClone; Ge Healthcare Life Sciences). After 3 days, the medium was replaced with fresh medium with the same concentration of M-CSF. BMDMs that were differentiated for 5–7 days were used for the experiment. For macrophage polarization, the BMDMs were incubated with 100 ng/ml LPS (Sigma; L2880) for 24 hours and then treated with sodium lactate (20 mmol/L) for another 24 hours, and 20 *μ*M SH-4–54 (Selleck Chemicals) and sodium lactate were added in the medium at the same time. For cell flow cytometry, the cells were digested with 0.25% trypsin from the culture plate, F4/80 was used to characterize macrophages, and pro-inflammatory was identified as TNF-*α*, IL-6, and IL-12, respectively.

### 2.8. Real-Time Quantitative RT-PCR (qRT-PCR) Analysis

Total RNAs were isolated by using TRIzol reagent according to the manufacturer's instructions (Invitrogen, CA, USA) and converted into cDNA using Thermo Scientific RevertAid RT Kit (Invitrogen, CA, USA). The mRNA levels of IL-6, IL-10, Arg-1, Fizz-1, and 18S were determined with SYBR Green PCR Kit (Qiagen, Venlo, Hilden, Germany). The target mRNA levels were normalized to the geometric mean of 18S mRNA levels. The primers for qRT-PCR were listed in Table 1.

### 2.9. Western Blot Analysis

Briefly, the proteins were isolated and resolved by electrophoresis on 10% SDS-polyacrylamide gels and transferred onto a polyvinylidene difluoride (PVDF) membrane. After 2 hours of incubation in Tris buffer with 0.1% Tween-20 (TBST) and 5% BSA, the membranes were incubated with specific primary antibodies for overnight. Primary antibodies, GAPDH, P-SATA3, DRP-1, and P-AKT, were obtained from Cell Signaling Technology (Boston, MA, USA). LC3B was obtained from Abcam. The membranes were subsequently incubated with HRP-conjugated goat anti-rabbit or goat anti-mouse IgG secondary antibodies for another 2 h. The activity of peroxidase retained on the blots was measured with enhanced chemiluminescence (ECL) detection reagents from GE Healthcare (Uppsala, Sweden) according to the manufacturer's instructions.

### 2.10. Statistical Analysis

Statistical analysis was performed using the GraphPad Prism 8 software. All data are presented as mean ± SEM. Normal distribution was tested using the Shapiro-Wilk test. Differences between two groups were compared by unpaired *t*-test. More than two groups were performed by one-way ANOVA, followed with a post hoc Tukey's multiple comparison test. All *P* values were two-tailed, and a *P* value <.05 was considered statistically significant.

## 3. Results

### 3.1. Sodium Lactate Exerted Cardioprotective Effects in a MI Mouse Model

**Figure 1:**
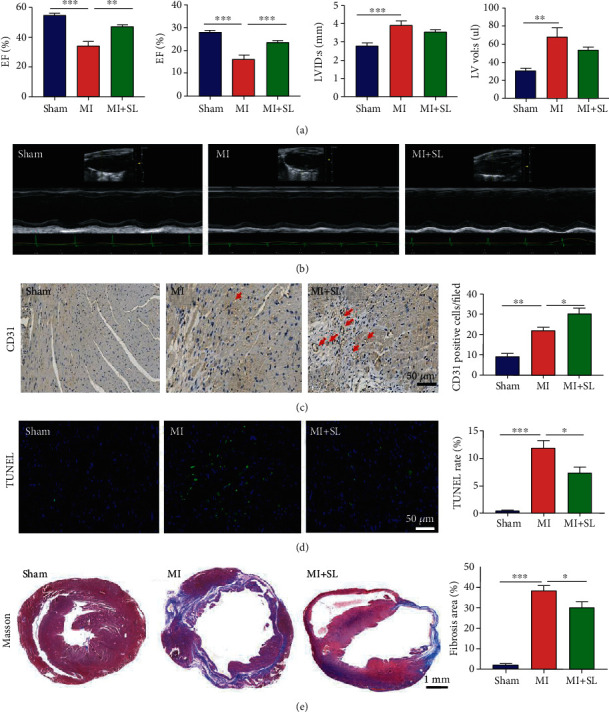
Sodium lactate treatment improved cardiac performance after myocardial infarction (MI). (a) Quantitative echocardiographic analysis. EF: ejection fraction; FS: fractional shortening; LVID: s: left ventricular internal diastolic at end-systolic dimension; LV vol: s: left ventricular volume at end diastole. (b) Typical echocardiographs in each group. (c) Immunohistochemical staining of CD31 showed that sodium lactate significantly increased new vessel formation in the ischemic area. (d) TUNEL staining was performed to estimate myocardial cell apoptosis. (e) Masson trichrome staining of the heart 2 weeks after the treatments and the quantitative analysis, scale bar, 1000 *μ*m. The values are expressed as the mean ± SEM. ^∗^*P* < .05, ^∗∗^*P* < .01, and ^∗∗∗^*P* < .001 (*n* = 5‐6).

First, we investigated cardioprotective effect of sodium lactate in myocardial injury. One day after the surgery, sodium lactate was administered by intraperitoneal injection for 14 consecutive days. Echocardiography was performed, and the examination showed that sodium lactate attenuated post-MI cardiac dysfunction by improving ejection fraction (EF) and fractional shortening (FS) (Figures 1(a) and 1(b)). Furthermore, the immunohistochemistry results reported that sodium lactate decreased cardiomyocyte apoptosis, restrained cardiac fibrosis, and increased new vessel density post-MI (Figures 1(c)–1(e)). These results indicated that sodium lactate could improve cardiac function after MI.

### 3.2. Sodium Lactate Attenuates Myocardial Infarction Injury via Enhancing Macrophage Polarization

**Figure 2:**
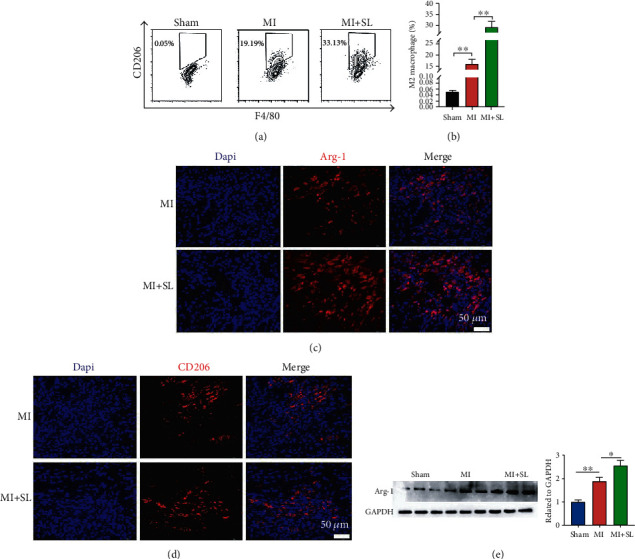
Sodium lactate increased the proportion of M2 macrophages post-MI. (a) Representative images of flow cytometric analysis of myocardial F4/80+CD206+ macrophages after saline or sodium lactate treatment for 3 days post-MI. (b) Analysis of the M2 macrophage expression in each group. (c) Immunofluorescence staining of M2 macrophage markers (CD206, red) in the ischemic area. (d) Immunofluorescence staining of M2 macrophage marker (Arg-1, red) in ischemic myocardial tissue. (e) Western blot analysis showed the expression of M2 macrophage marker Arg-1 after lactate or saline treatment post-MI. The values are expressed as the mean ± SEM. ^∗^*P* < .05, ^∗∗^*P* < .01, and ^∗∗∗^*P* < .001 (*n* = 3).

Previous studies found that lactate promoted macrophage polarization in tumor microenvironment [16], which inspired us to examine whether macrophage polarization participated in sodium lactate-mediated cardioprotection in MI. Additionally, immunofluorescence analysis of heart tissue showed an augmentation of Arg-1 and CD206 positive macrophage (M2-like) proportions compared with those of saline-treated MI mice (Figures 2(c) and 2(d)). Another, we measured Arg-1 protein expression in the ischemic heart tissues on day 3 after sodium lactate treatment. Notably, sodium lactate treatment markedly increased Arg-1 in the heart of MI mice compared with those of saline treatment (Figure 2(e)). Flow-cytometric analyses revealed that sodium lactate modulated macrophage polarization and the early inflammatory response following MI (Figures 2(a) and 2(b)).

### 3.3. Sodium Lactate Treatment Potentiated M2 Macrophage Polarization and Suppresses Proinflammatory Macrophage

**Figure 3:**
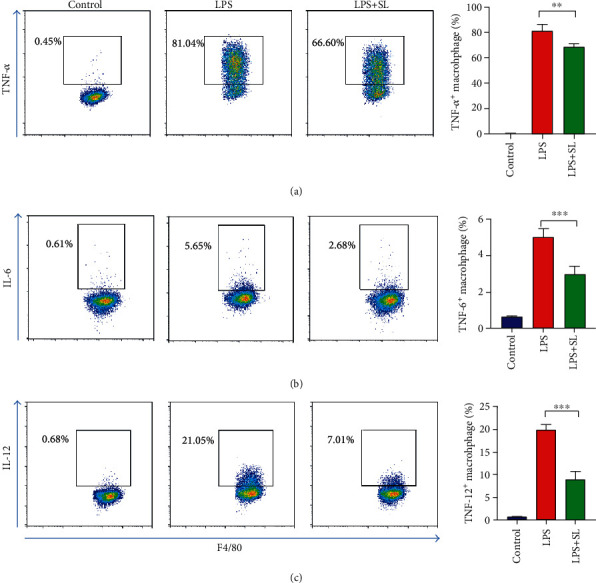
Sodium lactate treatment suppressed inflammatory macrophages in vitro. (a–c) Flow-cytometric analysis showed that sodium lactate repressed TNF-*α*+, IL-6+, and IL-12+ macrophages following LPS activation (*n* = 3). The values are expressed as the mean ± SEM. ^∗^*P* < .05, ^∗∗^*P* < .01, and ^∗∗∗^*P* < .001.

**Figure 4:**
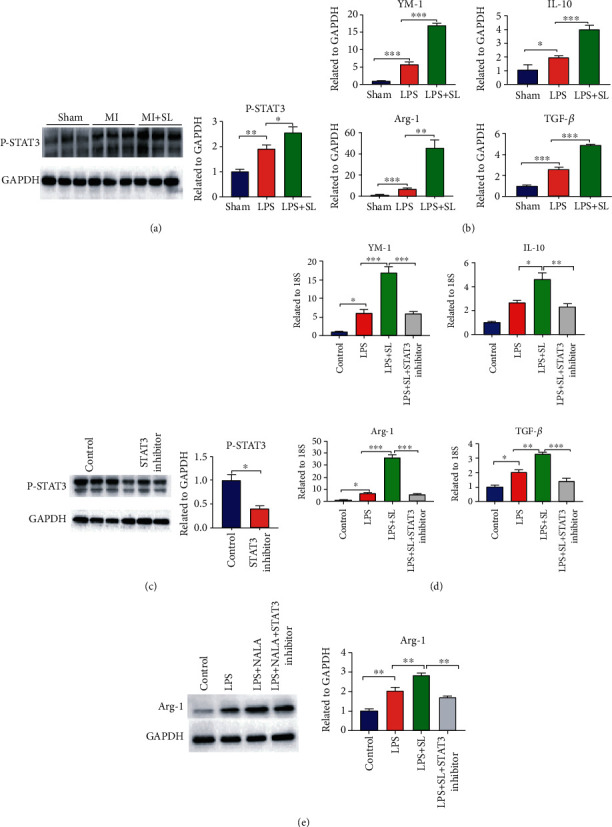
Sodium lactate accelerated macrophage polarization by enhancing P-STAT3 levels. (a) Western blot analysis reported that sodium lactate increased P-STAT3 expression after MI (*n* = 5‐6). (b) The q-PCR results showed Arg-1, IL-10, TGF-*β*, and YM-1 expression in LPS-stimulated BDMMs with or without sodium lactate treatment. (c) The STAT3 inhibitor significantly decreased P-STAT3 protein expression in BMDMs, as shown by Western blot. (d) The q-PCR results showed that sodium lactate promoted M2-like marker (Arg-1, IL-10, TGF-*β*, and YM-1) expression with LPS activated, which was suppressed by the STAT3 inhibitor. (e) protein expression of Arg-1 was evalutated by western blots (*n* = 3). The values are expressed as the mean ± SEM. ^∗^*P* < .05, ^∗∗^*P* < .01, and ^∗∗∗^*P* < .001.

Sodium lactate improved cardiac performance in an MI mouse model by increasing M2 macrophage proportion. Whether sodium lactate has the same effects in vitro was also explored. Thus, BMDMs were incubated with LPS (100 ng/ml) to induce macrophage polarization. To investigate the role of sodium lactate in macrophage polarization, we examined the expression of TNF-*α*+, IL-6+, and IL-12+ macrophages with flow cytometric analysis; also, the mRNA level of Arg-1, IL-10, YM-1, and TGF-*β* (M2 marker) in sodium lactate was stimulated. Sodium lactate decreased TNF-*α*+, IL-6+, and IL-12+ positive macrophage proportion (Figures 3(a)–3(c)) while increased Arg-1, IL-10, YM-1, and TGF-*β* expression (Figure 4(b)), suggesting that sodium lactate inhibited proinflammatory macrophages and promoted M2 macrophage polarization.

### 3.4. Sodium Lactate Enhanced Macrophage Polarization via P-STAT3

**Figure 5:**
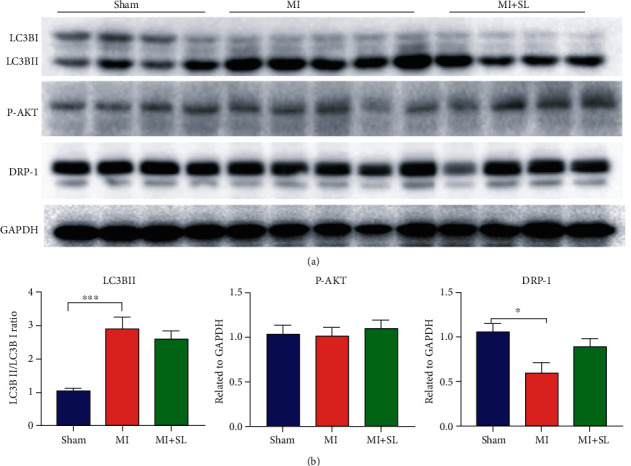
Sodium lactate did not affect LC3B, P-AKT, or DRP-1 expression and suppressed inflammatory macrophages in vitro. (a) Representative Western blot images showing LC3B, P-AKT, and DRP-1 post-MI with lactate or saline treatment for 3 days (*n* = 5‐6). (b) Analysis of the protein-related expression of LC3B, P-AKT, and DRP-1 in MI mice with or without sodium lactate.

To elucidate the underlying mechanisms, LC3B, P-AKT, and dynamin-1-like protein (DRP-1) in ischemia heart tissue were examined (Figures 5(a) and 5(b)), and these proteins were not affected by sodium lactate in the context of MI. Furthermore, we examined the effects of sodium lactate on the expression levels of phosphorylation-signal transducer and activator of transcription 3 (P-STAT3) in a MI mouse model. Interestingly, P-STAT3 expression was increased by intraperitoneal injection with sodium lactate (Figure 4(a)). To verify P-STAT3 is a target of sodium lactate, a STAT-3 inhibitor was used, and we found that the STAT-3 inhibitor successfully suppressed P-STAT3 expression in BMDMs (Figure 4(c)). Then, the STAT3 inhibitor was administered with sodium lactate to LPS-stimulated BMDMs. As expected, the STAT-3 inhibitor suppressed P-STAT3 upregulation and reversed the effect of sodium lactate on macrophage polarization (Figures 4(d) and 4(e)). In summary, our results demonstrated that sodium lactate enhanced macrophage polarization through the P-STAT3 signaling pathway.

## 4. Discussion

Recently, increasing number of studies has begun to show the new and unexpected biological functions of lactate, which was previously considered as a by-product. Until now, lactate has been recognized as a major carbon source for cellular metabolism and as a signaling molecule both in normal, chronically inflamed tissues and cancerous tissues [19–23]. Interestingly, Nolt et al. found that hypertonic sodium lactate fluid protected against cardiac dysfunction during sepsis and simultaneously reduced inflammation [24]. Another study also reported that an infusion of half-molar sodium lactate could improve cardiac performance in acute heart failure patients without any detrimental effects on organ function. Moreover, studies have shown that postconditioning with lactate-enriched blood provides potential cardioprotection in MI patients with ST-segment elevation who undergo primary percutaneous coronary intervention [25–27]. Accumulating evidence suggests that lactate may be a promising treatment for heart disease [28]. Here, we used sodium lactate to treat mice after MI, and two weeks later, myocardial infarction mice that had been treated with sodium lactate exhibited improvements in EF and FS compared with those in the vehicle group and significantly increased microvessel density in the border zone. We determined the percentage of M2 macrophages in the total cell population by using immunofluorescence and flow cytometric analysis of MI heart tissue, and the results revealed that sodium lactate increased the M2 macrophage proportion three days after MI. Additionally, in vitro, sodium lactate also suppressed IL-6+, IL-12, and TNF-*α*+ macrophages in LPS-stimulated BMDMs and induced robust expression of IL-10, Fizz-1, Arg-1, and TGF-*β*, indicating that sodium lactate promoted proinflammatory macrophages to polarize to anti-inflammatory macrophage. To further verify the mechanism of sodium lactate associated in macrophage polarization, LC3B, P-AKT, and DRP-1 were measured and the levels of those protein were no significantly changed. The results manifested that LC3B, P-AKT, and DRP-1 were not involved in sodium lactate-mediated macrophage polarization in MI. Furthermore, we found that sodium lactate enhanced the expression of P-STAT3 in ischemic heart tissue. Based on these results, we concluded that P-STAT3 may be the target of sodium lactate in MI. STAT3, in one of the seven STAT members, has increasingly gained attention due to its significant roles in diverse biological processes, including cell proliferation, cell survival, inflammation, immunity, and angiogenesis and also plays an important role in inducing macrophage polarization. STAT3 has been demonstrated to play roles in several cardiovascular diseases, including MI, arteriosclerosis, cardiac hypertrophy, and heart failure [29–31]. A previous study demonstrated the involvement of STAT3 signaling in lactate-mediated TAM polarization [15]. Here, we found that P-STAT3 participated in LPS-induced M2 macrophage polarization. Moreover, sodium lactate also enhanced P-STAT3 levels after MI, suggesting that P-STAT3 may be the key target by which sodium lactate mediates macrophage polarization. Notably, the function of sodium lactate-activated M2 macrophages was abrogated by inhibiting STAT3 signaling. These data suggested that sodium lactate is involved in macrophage M2 polarization by activating STAT3 signaling pathway, which may be beneficial for the development of novel and effective therapeutic approaches for MI.

However, there are still limitations in our study. The microenvironmental cues that drive macrophage polarization in MI and which specific M2 macrophage phenotype is involved in sodium lactate-mediated macrophage polarization also remain undetermined. Based on these findings, further investigations are highly required to demonstrate the phenotype and functions of macrophages in homeostasis and MI, as well as the detailed mechanisms of macrophage polarization in MI [32, 33]. Taken together, our results suggest that sodium lactate improves post-MI healing and cardiac function by favoring macrophage polarization, especially by increasing P-STAT3 pathway. Thus, we believe that sodium lactate should be further researched as a potential therapy for MI.

## Figures and Tables

**Table 1:** 

	Forward primer	Reverse primer
Arg-1	CTCCAAGCCAAAGTCCTTAGAG	AGGAGCTGTCATTAGGGACATC
Fizz-1	GGTCCCAGTGCATATGGATGAGACCATAG	CACCTCTTCACTCGAGGGACAGTTGGCAGC
TGF-*β*	CGCCTCTATGAGAAAACC	GTAACGCCAGGAATTGT
IL-10	GCTCTTACTGACTGGCATGAG	CGCAGCTCTAGGAGCATGTG
18S	AGTCCCTGCCCTTTGTACACA	CGATCCGAGGGCCTCACTA

## Data Availability

The data used to support the findings of this study are available from the submitting author (zjl094@126.com) upon request.

## Abbreviations

MI: Myocardial infarction
LPS: Lipopolysaccharide
BMDMs: Bone marrow-derived macrophages
SL: Sodium lactate
P-STAT3: Phosphorylation-signal transducer and activator of transcription 3
Arg-1: Arginase-1
IL: Interleukin
DRP-1: Dynamin-1-like protein
P-AKT: Phosphorylation threonine-protein kinases.
